# Adherence and cost in multiple sclerosis patients treated with IM IFN beta-1a: impact of the CARE patient management program

**DOI:** 10.1186/s12883-015-0426-x

**Published:** 2015-09-22

**Authors:** Zaza Katsarava, Birgit Ehlken, Volker Limmroth, Kirsi Taipale, Sarita Noemi Patel, Gabriele Niemczyk, Karin Rehberg-Weber, Colin Wernsdörfer, for the C.A.R.E. Study Group

**Affiliations:** Department of Neurology, Evangelic Hospital Unna, Holbeinstr. 10, 59423 Unna, Germany; Department of Neurology, University Hospital Essen, Essen, Germany; IMS HEALTH, Munich, Germany; Department of Neurology, Cologne City Hospitals, University of Cologne, Cologne, Germany; Biogen GmbH, Ismaning, Germany

**Keywords:** Patient management program, Adherence, Cost in MS

## Abstract

**Background:**

Disease modifying treatments (DMT) for MS such as interferon beta (IFNβ) have been shown to reduce the risk for disease progression. Therefore adherence to treatment is essential for treatment outcome.Here we want to evaluate if participation in a patient management program (PMP) improves adherence to DMT as well as health and cost outcomes associated with MS.

**Methods:**

In this open-label multicentre prospective observational study, German MS patients treated with once weekly intramuscular (IM) IFNβ-1a (Avonex ®), were offered participation in a PMP and followed for up to 12 months. The PMP included injection trainings, support and quarterly visits for up to 12 months after initiation of therapy. Utilisation of health care services was evaluated.

The primary endpoint was to evaluate the direct and indirect cost associated with MS from payer, patient and societal perspective, in patients who participate in the PMP. Secondary endpoint was the clinical outcome in patients who participate in the PMP (differentiated in adherent versus non-adherent patients).

**Results:**

In total 731 patients (mean age: 38.2, 73.7 % female) were enrolled, 640 (88 %) were observed for twelve months. After six months 34 % of patients had participated in the PMP continuously and 21 % temporarily; 39 % had not participated. After twelve months, the proportions of participants were: 37 % continuously and 19 % temporarily; 40 % had not participated.

After 6 months, mean reduction in cost per patient in the participants group (€ 2151) was almost twice as high as the cost reduction amongst non-participants (€ 1131).

After twelve months, the annual relapse rate was reduced by 58 % compared to baseline in both the participant and non-participant groups.

**Conclusions:**

In a real-world-setting, participation in a patient management program was associated with improved medication adherence and lower total MS-related direct and indirect cost over time.

## Background

Multiple sclerosis (MS) is an inflammatory and progressive disease of the central nervous system with a heterogeneous clinical course. MS usually begins with an acute inflammatory demyelinating episode (clinically isolated syndrome, CIS) turning to relapsing-remitting MS (RRMS) characterized by discrete acute attacks of worsening neurological function (relapses) that are followed by partial or complete recovery (remission). Residual disability may accumulate over time and many patients with RRMS develop secondary progressive MS, which is characterized by progressive neurological decline [1].

Therapeutic options in MS include treatment of acute relapses, immunomodulating disease-modifying drugs (DMD), and symptomatic therapy. A large number of randomized clinical trials showed that DMDs reduce relapse rates, decrease the occurrence of new lesions on magnetic resonance imaging (MRI), and reduce disease progression in patients with MS [2–7]. Apart from recently introduced oral drugs, first-line DMDs are self-administered parenterally (subcutaneously or intramuscularly) on a regular basis by the patient and are given daily to once weekly, depending on the product.

Although treatment efficacy has been established, adherence and persistence to pharmacotherapy in the long-term remains one of the main challenges and is a key factor in predicting long-term outcomes. Continuous use of DMTs provides the greatest benefit by preventing relapses and delaying disease progression [8, 9]. Furthermore, DMT-adherent patients experience higher quality of life [10, 11], show reduced absenteeism [9], require less health-care resources and cause lower medical cost [9, 12]. Published adherence rates in RRMS patients are 41–88 % depending on study and definition of adherence [10, 11, 13, 14]. Several studies comparing adherence rates for routine therapy using different DMTs reported the highest values for once-weekly interferon beta-1a (IM IFNb-1a) (79–85 vs. 49–78 % for other DMTs) [10, 11]. The risk of discontinuation of therapy has been shown to be highest early after treatment initiation, i.e., within the first 6 months to 2 years [15, 16]. Optimal adherence to immunotherapy is essential for MS patients to achieve the full long-term benefit of their therapies. However, patients with MS face several barriers to optimal treatment adherence. Multiple therapy-related factors such as adverse events (e.g., flu-like symptoms, injection-site reactions), efficacy concerns or injection-related reasons can result in non-adherence. Additionally, adherence may be affected by the patients’ cognitive impairment, depression, inadequate expectations, knowledge deficits or the patient´s attitude towards himself and the disease. Other factors such as social/family support, relationship between patient and healthcare provider, and socio-economic factors were also identified as factors that compromize adherence [10, 11, 17, 18].

Optimizing adherence to MS therapy is an important therapeutic goal. The substantial economic burden associated with MS results from direct medical cost associated with healthcare and disability-related resource utilization as well as indirect cost related to reduced productivity [19]. Non-adherence has been shown to be associated with higher MS-related medical cost [12]. Therefore improving poor adherence could result in benefits for patients, payers and society. Strategies to enhance adherence to DMTs include managing patient expectations and adverse events, educating patient and family and importantly establishing a good relationship between patients and treating physicians [17]. Adherence to therapy can also be improved by providing the patient with a supporting network. Specialized MS nurses can give valuable assistance advising the patient on the management of symptoms, side-effects or injection anxiety. Many studies have reported the potential benefit of patient management programs (PMPs) in other therapeutic areas such as diabetes, coronary artery disease or asthma [20].

The context of PMPs enables continuous patient education and consistent confirmation of treatment benefit [21]. A structured PMP with specialized nurses or skilled healthcare workers supporting the patients after starting immunotherapy may have a substantial impact on MS patient outcomes. Therefore, the patient management program MS-CARE has been introduced in Germany. It offers personalized injection training, regular telephone-based or personal counselling, patient education services, and exchange of experiences.

The aim of the present study C.A.R.E.. (Does personal Care for Avonex® patients with RRMS increase Effectiveness of treatment?) was to investigate the impact of a PMP on patients’ adherence, and clinical and economical outcomes among patients with MS treated with once weekly IM IFNb-1a.

## Methods

C.A.R.E. was an open-label non-interventional, observational, prospective, multicenter study conducted in Germany between February 2009 and December 2010, investigating the impact of a structured PMP on patients’ treatment adherence and associated health and economical outcomes. The patients were observed for 12 months in a real-world clinical practice setting. Adult patients with a clinically isolated syndrome (CIS), or with a diagnosis of RRMS who had received a prescription for once weekly IM IFNb-1a for at least one month before inclusion were included in the non-interventional study.

The study was conducted in agreement with the International Conference on Harmonisation (ICH) and Good Clinical Practice (GCP) guidelines and followed Declaration of Helsinki recommendations. Prof. Dr. Karl H. Jakobs, representative for the ethics commission of the Medical Faculty of the University Duisburg-Essen (Ethik-Kommission, Robert-Koch-Str. 9–11, 45147 Essen, Germany), approved the study. All patients provided written informed consent.

### Data collection

Data collection was based on physician and patient questionnaires (administered to physicians every 3 months, and to patients every 6 months). At study initiation (visit 1) physicians collected the patients’ demographic data, medical history, date of MS diagnosis, prior treatment with other DMTs, current disease characteristics, and the type of pharmaceutical formulation used for IM IFNb-1a therapy. The patients also reported if they self-injected IM IFNb-1a or required assistance. At the following quarterly visits, physicians documented the IM IFNb-1a treatment status and the course of disease (relapses, affected functional systems, steroid therapy, and hospitalizations). The patients’ adherence was rated on a 10-step scale (1 = excellent, 10 = very poor) as judged by the physician. Patients with ratings of 2 or lower were considered adherent since adherence of at least 80 % is a widely accepted adherence criterion [22, 23].

At study initiation and after 6 and 12 months, respectively, the patients completed a patient survey. This questionnaire included questions concerning professional life, general practitioner and specialist visits, examinations, hospital care (inpatient, outpatient), rehabilitation, home care, expenses for medication (non-prescription drugs, analgesics, nutritional supplements), investments, and acquisitions of aids and appliances. The patient questionnaires also provided information about participation in the PMP.

At visit 1, the patients were offered participation in the PMP MS-CARE. Patients who participated in the PMP are denoted “participants” and those who did not, “non-participants”. The PMP was based on individual patient education by MS nurses. They provided consistent education on MS as disease, treatment, and motivation for treatment. The nurses performed injection trainings within the first weeks of therapy and provided advice on improving injection technique and tolerability. Following this initial training, the same nurse supported the individual patient, provided motivation, and monitored adherence throughout the study. During regular personal meetings or by telephone, the nurses provided general advice and helped improve injection handling. Information and service material was provided upon request.

In the economic analyses, cost was evaluated from the payer (statutory health insurance (SHI), statutory pension insurance), patient, and societal perspectives. Direct cost from payer perspective include cost for physician visits and medical services in the outpatient setting, hospitalisations, inpatient and outpatient rehabilitation measures, and nursing home. Cost for IM IFNb-1a was not considered in the analysis. Direct cost from patient perspective included co-payments (e.g., for wheelchair, medical devices, glasses), expenses for non-prescription medication and transportation, and modifications of home and car (e.g., elevator). Indirect cost from societal perspective included productivity losses due to workdays lost. For the calculation of indirect cost, the number of days absent from work was multiplied with the average loss of productivity per day. The economic evaluation was done in accordance with German recommendations for health economic evaluations [24].

Unit cost of physician visits and medical services at office-based practices was derived from the official German physicians` fee schedule (“Einheitlicher Bewertungsmaßstab”, EBM, 2011) [25]. Unit cost for visits to other specialists (ergotherapy, physiotherapy, alternative medicine) was taken from the AOK (a statutory health insurance company) homepage [26] and the alternative practitioners homepage [27]. Unit cost for hospitalisations due to MS was based on case related allowances using German diagnosis related groups (G-DRG) [28, 29]; an average base rate of € 2964 was used for calculation. Since official nationwide unit cost for outpatient rehabilitation and inpatient rehabilitation was not available, unit cost per day was taken from homepages of individual rehabilitation hospitals. Based on gross income data and the number of persons in paid employment, the monetary value of productivity loss per day (€133.85) for employed persons was calculated [30]. Cost from the patient perspective was directly derived from patients’ questionnaires.

### Statistical analysis

Continuous data were analysed by descriptive statistics (number, mean, standard deviation, minimum, 5th, 25th, 75th, and 95th percentile, maximum; for cost data percentiles are not shown). Percentages given in the Table 1 are based on the proportions of patients with available data for the respective item. For categorical parameters, absolute and relative frequencies were calculated. Relative frequencies refer to the number of patients with evaluable data. The number of patients with evaluable data may vary between different analyses.

**Table 1:** Demographic data and baseline characteristics of the study population (all patients, documentation in physician questionnaires)

Patient population		
Patients enrolled [n]		731
Therapy discontinuation:	No [n]	614 [84 %]
	Yes [n]	117 [16 %]
Duration of observation in C.A.R.E. [days], mean/median (range)		309/351 (8–699)
Baseline characteristics		
Age [years], mean/median (range)		38.2 (+/−10,8)/ 39 (17–69)
Gender, female [%]		73.7
# Relapses 12 months prior to baseline [n], mean/median (range)		1.3/1.0 (0–6)
Duration of MS before baseline [years], mean/median (range)		5.2/2.0 (1–41)
Prior MS therapies [%]		
	No	485 [66.4 %]
	Yes	245 [33.6 %]
	Missing	1

## Results

Data from 731 patients with CIS or RRMS on once weekly IM IFNb-1a were included in the analysis. Demographic data and baseline disease characteristics of the total study population are shown in Table 1.

Among the included patients, 73.7 % were female, the mean age at baseline was 38.2 ± 10.8 years. Patient medical history revealed that the patients had on average 1.3 relapses (range 0–6) in the year before inclusion in the study. Mean duration of MS prior to baseline visit was 5.2 years (range 1–41 years).

The mean adherence rating was “good”, i.e., 2.0 (±1,9) on an ordinal scale from 1 (“excellent”) to 10 (“very poor”) as judged the treating neurologist.

**Fig. 1:**
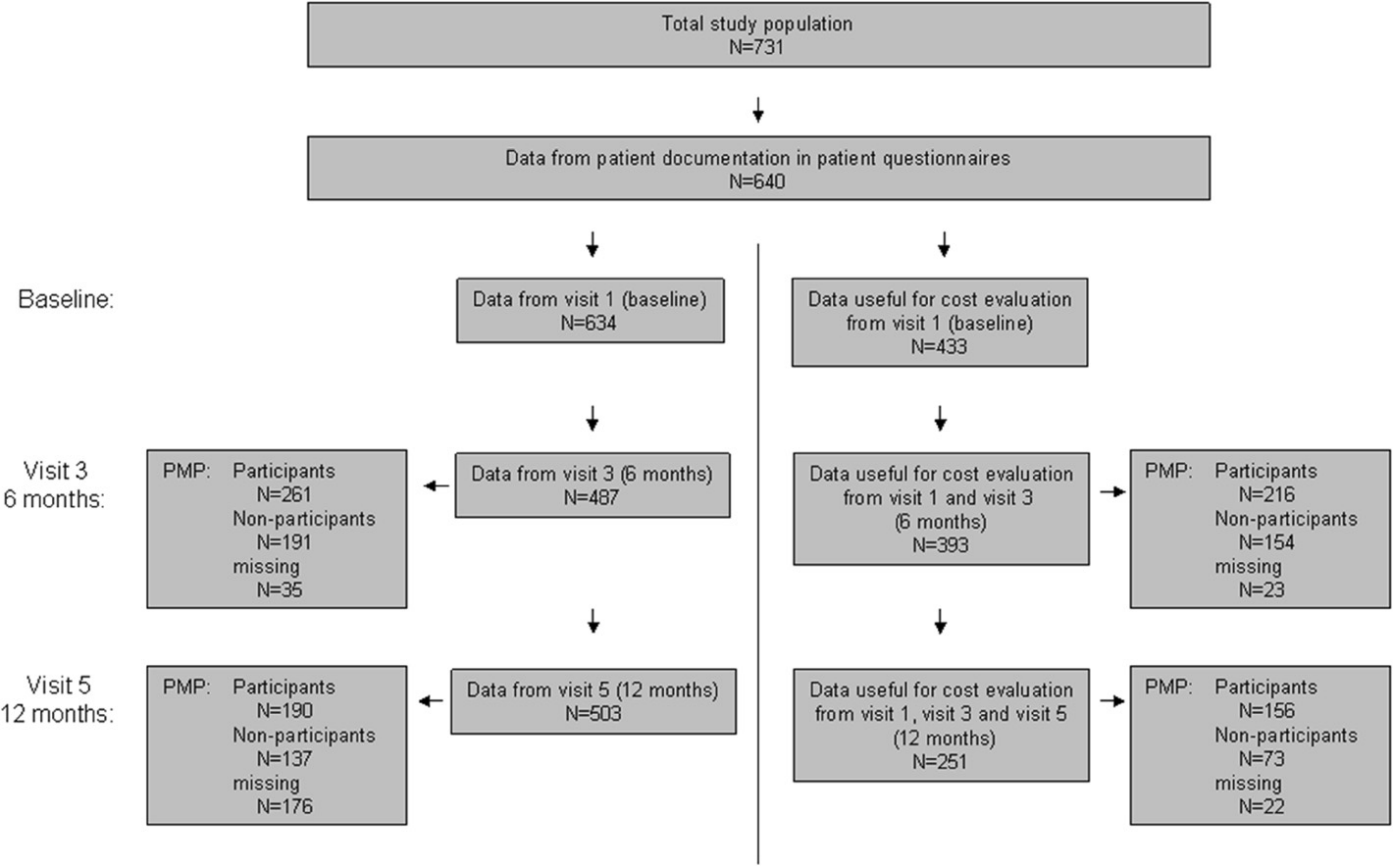
Study overview: flow chart representing the patient flow through consecutive study visits

For several subgroup analyses, e.g., impact of the PMP on adherence, effectiveness or cost, data from the patient questionnaires were used. An overview of the numbers of patients with evaluable data from the questionnaires at different time points is given in Fig. 1. In total, the patient questionnaire was completed by 88 % (640/731) of patients. At visits 1 (baseline), 3 (at 6 months), and 5 (at 12 months), respectively, 99 % (634/640), 76 % (487/640), and 79 % (503/640) of patients completed the questionnaire. Because of the non-interventional character of the study, PMP participation was voluntary. At visit 3 (6 months) 54 % (261/487) of patients who had completed the questionnaire indicated that they had participated in the PMP while 191 patients (39 %) did not participate (data missing for 35 patients). At 12 months, 38 % (190/503) patients had further participated in the PMP whereas 137 patients (27 %) did not participate (data were missing for 176 patients).

Data for cost evaluation at visit 1 were available for 68 % of patients (433/640), at both visit 1 and visit 3 for 61 % (393/640), and data from all three visits (12 months period) were available for 39 % (251/640).

The proportion of patients with available data for cost analysis at visits 1 and 3 (six months period) who had participated in the PMP was 55 % (216/393); 40 % (154/393) were non-participants (data were missing for 23 patients).

The mean age of documented PMP participants was 38.2 years at visit 3 and 38.1 years at visit 5 (12 months) (Table 2). Participants were slightly younger than non-participants (mean age 38.2 vs. 39.2 years, and 38.1 vs. 40.0 years at 6 and 12 months, respectively). The proportion of women was slightly higher in the PMP participant group (76.2 % at 6 months and 77.9 % at 12 months) versus non-participants (70.5 % and 75.7 %). There was no significant difference in mean annual relapse rate and proportion of prior MS therapies between participants and non-participants.

**Table 2:** Demographic data and baseline characteristics of PMP participants and non-participants (*n* = 452 at visit 3 (6 months) and *n* = 327 at visit 5 (12 months), patient questionnaires)

		Participation in PMP			
		Months 0-6		Months 7-12	
		Yes	No	Yes	No
Patients [n]		261	191	190	137
Age, mean/median [years]		38.2/38.0	39.2/40.0	38.1/37.5	40.0/41.0
Gender, female [%]		76.2	70.5	77.9	75.7
Number of relapses during 12 months prior to baseline [n]		1.2 (0.8)	1.2 (1.0)	1.2 (0.8)	1.2 (0.9)
Prior MS therapy [%]	No	32.6	31.1	34.7	29.4
	Yes	67.4	68.9	65.3	70.6

### Adherence

**Fig. 2:**
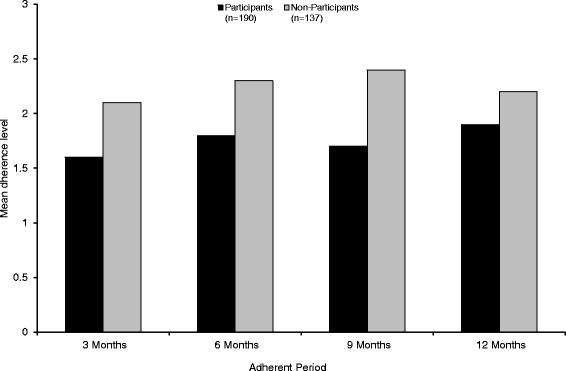
Neurologists´ rating of patients’ adherence (mean adherence rating) on a 10-step adherence scale (1 = excellent; 10 = very poor); PMP participants versus non-participants

Overall treatment with once weekly IM IFNb-1a was associated with high physician-reported adherence during the 12 months observational period. The mean adherence rating over all patients was 1.9 (“good”) at visit 2 after 3 months and 2.0 (“good”) at all following visits (data not shown). After 12 months, 96.3 % of patients with available data (395/410) were still adherent to once weekly IM IFNb-1a therapy. The formulation (pre-filled syringe or lyophilised) of IM IFNb-1a did not have any impact on adherence (data not shown). Participating in the PMP showed a trend towards higher adherence rating by the treating physician (Fig. 2). Participants were more often rated as adherent (a rating of 2 corresponds to 80 % adherence) by the treating physician than non-participants. However, the difference was not statistically significant (84.2 % vs. 79.6 %; *p* = 0.3058).

### Course of disease

The mean annual relapse rate (ARR) of the total patient population decreased from 1.3 during the year preceding study entry to 0.4 during the 12 months of study. A total of 71 % of the patients remained free of relapses during the study (data not shown). There was no significant difference in the mean ARR after 12 months between PMP participants and non-participants (*p* = 0.8976). Both participants and non-participants experienced a mean of 0.5 relapses within 12 months which corresponds to a 58 % relative reduction of relapse rates in both groups (Table 3).

**Table 3:** Annual relapse rate (ARR) and relative reduction of relapse rate

Mean ARR	Participants (*n* = 190)	Non-participants (*n* = 137)
During12 months before baseline	1.2	1.2
From baseline to month 12 of study	0.5	0.5
Relative reduction of relapse rate	58 %	58 %

### MS-related cost

**Fig. 3:**
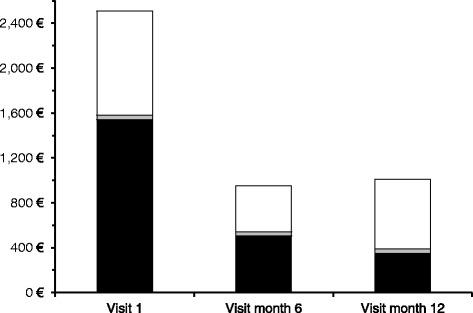
Mean total cost per 6 months per patient from societal perspective [€] 6 months before visit 1, and at visits 3 (month 6) and 5 (month 12); patients with available data for all visits (n = 167)

Data for the economic analysis were derived from the patients’ questionnaires. At baseline, data for cost evaluation were available from 433 patients. Only patients with documentation at least in visits 1 (baseline) and 3 (after 6 months; *n* = 393) were included in the analysis. After 12 months, this sub-population included 251 patients. Complete data for cost analysis from societal perspective for all three visits were available for 167 patients. The total cost from societal perspective for the six months period prior to study enrolment was € 2511 ± 3292 per patient. The cost from the payers’ perspective accounted for 61 %, from the patients’ perspective for 2 % and the cost for loss of productivity accounted for 37 % of the total cost. Within 6 months after study inclusion, the total cost from societal perspective was reduced to € 951 ± 1967 per patient (Table 4, Fig. 3). The difference is statistically significant (*p* < 0.0001). This reduction was mainly due to markedly lower cost for hospital stays (€ 967 to € 56) and to a lesser extent to reduction in cost for visits to other specialised health care professionals (€ 69 to € 31), examinations (€ 207 to € 68) and stays at a day-care clinic (€ 76 to € 11). Cost from the payers’ perspective dropped to one third of baseline cost, indirect cost was reduced to less than half after 6 months whereas cost from the patients’ perspective remained unchanged. The reduction in total cost persisted at twelve months (€ 1,009 ± 2,780).

**Table 4:** Total cost per patient [€] from the societal perspective 6 months before visit 1, visit 3 (month 6) and visit 5 (month 12); patients with data at baseline, 6 months and 12 months of study (*n* = 167, cost data without missing data for all three visits), *p* < 0.0001

	Baseline visit					Visit at month 6					Visit at month 12				
	Mean	SD	Median	Min	Max	Mean	SD	Median	Min	Max	Mean	SD	Median	Min	Max
Direct cost, payer perspective	1,540	2,124	362	0	11,145	506	1,275	120	0	7,071	350	971	121	0	7,770
Visits, other specialists	69	69	0	0	290	31	46	0	0	222	37	56	0	0	273
Physician visits	66	183	0	0	1,369	51	140	0	0	869	70	172	0	0	1,201
Examinations	207	199	186	0	1,512	68	100	0	0	623	72	95	6	0	486
Hospital stays	967	1,632	0	0	6,212	56	414	0	0	3,106	56	414	0	0	3,106
Daycare clinic	76	323	0	0	3,252	11	69	0	0	542	11	69	0	0	542
Inpatient rehabilitation	149	822	0	0	5,893	289	1,173	0	0	7,071	99	759	0	0	7,071
Outpatient rehabilitation	6	44	0	0	416	0	0	0	0	0	6	54	0	0	555
Nursing home stay	0	0	0	0	0	0	0	0	0	0	0	0	0	0	0
Direct cost, patient perspective	40	98	0	0	730	35	78	0	0	585	37	89	0	0	700
Indirect cost (loss of productivity)	931	2,125	0	0	20,078	410	1,288	0	0	12,047	621	2,421	0	0	24,628
Total cost	2,511	3,292	825	0	22,013	951	1,967	179	0	12,296	1,009	2,780	251	0	24,970

### Relationship between PMP participation and cost

**Fig. 4:**
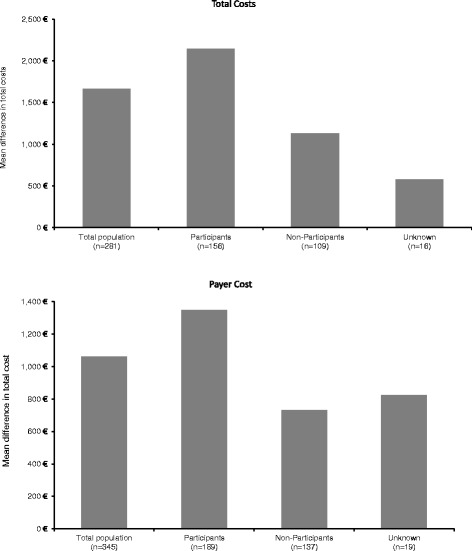
Mean reduction in cost per patient after 6 months on study versus 6 months before baseline; PMP participants vs. non-participants. **a** Mean reduction in total cost per patient from societal perspective (281 patients with cost data available for visits 1 and 3). **b** Mean reduction in cost per patient from payers’ perspective (345 patients with cost data available for visits 1 and 3)

To evaluate the impact of PMP participation on MS related cost data at visit 1 and 3 were analysed from the societal perspective (*n* = 281) and from the payers’ perspective. (*n* = 345). After 6 months, the mean cost reduction per patient from the societal perspective was almost twice as high in participants (€ 2,151) versus non-participants (€ 1,131) (Fig. 4a). These reductions are mostly due to a reduction in cost from the payers’ perspective and to a lesser extent to reduced loss of productivity. From the payers’ perspective alone (Fig. 4b), cost as reduced by € 1,322 in the participants group and by € 731 among non-participants. These savings resulted mainly from fewer hospital stays, examinations, and physician visits. Data indicated that reduction in both total cost and insurance-covered cost (payers’ perspective) was greatest in patients participating in the PMP. However, for participants MS-related cost at baseline had been higher than for non-participants (data not shown).

### Relationship between treatment adherence and cost

**Fig. 5:**
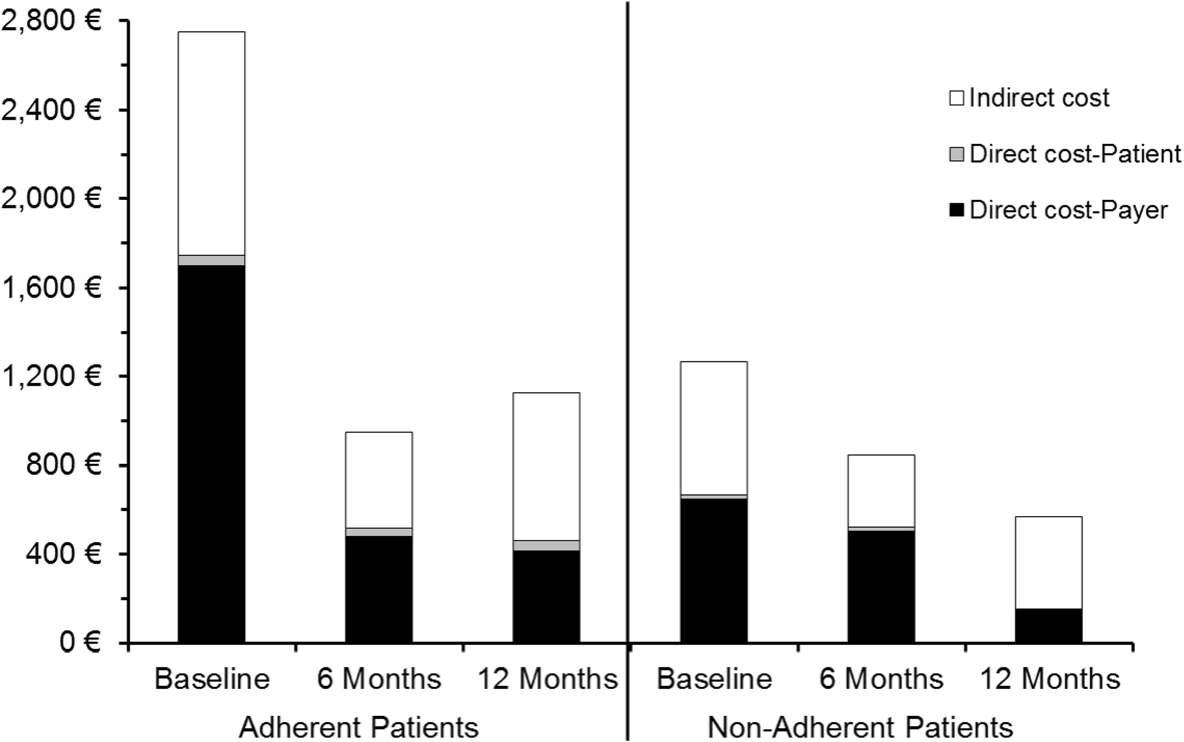
Mean total cost per patient from the societal perspective accrued in 6-months intervals before and during study (167 patients with cost data at visits 1, 3 and 5); adherent vs. non-adherent patients

The cost reduction after start of the study as compared to the six-month period before is shown for adherent and non-adherent patients, respectively (Fig. 5). Data of 166 patients at baseline and at 6 and 12 months were available for analyzing cost stratified for adherence. Of this subpopulation of patients, 83 % were adherent and 17 % non-adherent. Total cost from the societal perspective in the 6 months prior to study start had been €2,748 ± 3,451 per patient for the adherent patients and € 1269 ± 2030 for non-adherent patients. These cost was reduced to € 951 ± 1,934 and € 845 ± 2,109, respectively, within 6 months. Thus, the cost reduction was somewhat more pronounced in the adherent group than in the non-adherent group. However, in the 6 months before study entry the cost had been higher in the adherent subgroup.

At visit 3 after 6 months, cost was comparable between adherent and non-adherent patients. The reduction of mean total cost per patient was maintained for 12 months for both adherent (reduced by € 1,619) and non-adherent patients (reduced by € 700).

## Discussion

This study aimed to provide data on the impact of a treatment-accompanying PMP on patients’ adherence and associated clinical and economical outcomes among patients with MS treated with IM IFNb-1a in a real-life practice setting using physician-based reports and patient questionnaires.

The overall adherence rate found in this observational study was approximately 80 % (corresponding to a mean adherence rating of 2.0). Patients who participated in the PMP were more adherent than non-participants to once weekly IM IFNb-1a treatment after 12 months as rated by the treating physician (84 vs. 79 %, difference statistically not significant). This suggests a positive effect of the patient support in the patient management program. Data from a previous study show that medication adherence and persistence to MS therapy improved among participants in a PMP but deteriorated among non-participants [31]. The observed adherence rate is in the range of several previous retrospective and prospective studies showing adherence rates between 41 and 88 % [10, 11, 14].

However, a comparison of adherence rates between different studies is difficult due to different approaches in measuring adherence. In this non-interventional study, adherence was rated by the treating physician using personal judgement compared to methods based on direct patient interviews concerning the injection related behaviour in a certain time period, prescription patterns or medication possession ratios [9–11, 18, 32, 33]. Data from a Spanish study indicate that only a minority of patients informs the neurologist when they skip injections or interrupt DMT treatment [13]. The physician’s perception may therefore differ from the patient’s estimate of adherence. The findings of the global “MS Choices Survey” suggested that physicians may underestimate the levels of adherence among their patients [34]. Interestingly we noted no significant difference in adherence between patients using the pre-filled syringe or the lyophilisate. Possibly adherence may be further improved by advances in delivery technology such as the use of autoinjectors. In a phase IIIb-study [35], patients preferred a pen device for administering once weekly IM IFNb-1a versus a conventional syringe. At the time when the present study was conducted the pen device was not yet available. Our study showed that PMP participants were more often adherent than non-participants. These results suggest that the PMP may have a positive impact on patient adherence and might consequently bring about a higher treatment benefit.

During the 12-month study period, both participants and non-participants in the PMP showed a 58 % relative risk reduction in the ARR. However, due to the lack of control groups, conclusions about efficacy cannot be reliably derived from the data. Furthermore, the observation period of 12 months is too short to detect any relevant long-term effects.

One major objective of the present observational study was to evaluate the effect of the PMP on economical outcomes. In the total study population, the mean reduction of cost in 6 month after versus before the initiation of IM IFNb-1a treatment was € 1066 from payers’ perspective and € 1502 from the societal perspective. When stratifying MS-related cost by participation in PMP, participants showed a higher decrease of mean total MS-related cost after 6 months than non-participants (reduction by € 2151 vs. € 1131). Total MS-related cost as well were reduced more strongly in adherent vs. non-adherent patients (€ 1,619 vs. € 700, respectively). A retrospective cohort analysis by Tan et al. in the US demonstrated that mean MS-related medical cost decreased by € 197 among participants and increased by US$ 1536 among non-participants. Participants utilize fewer medical services including hospitalizations, emergency department visits and unscheduled physician office visits [31].

Non-adherence has been related to higher cost for hospitalisations, physician and emergency department visits in another retrospective study [9]. The reductions in cost observed in our observational study were mainly caused by lower cost for hospital stays indicating a successful improvement of clinical outcomes as the hospitalization rate serves as indicator for severe MS exacerbations [9]. This may also explain the observed reduction in total MS-associated cost in non-adherent patients and non-participants. Both PMP participants and adherent patients in our study had caused higher MS-associated cost prior to study entry. These patients may have more severe MS than non-participants or non-adherent patients and required more hospital stays. Higher baseline cost was also reported in previous studies [9, 31]. One further explanation for higher baseline cost in patients turning out adherent or participating in a PMP might be that they generally tend to adhere to visit or examination schedules more stringently. Furthermore they more often consult physicians and other healthcare providers for a second opinion.

Our study is subject to several limitations. First, randomization of the intervention (i.e., participation in the PMP) was not performed due to the observational nature of the study. Therefore, participation in the PMP may have been biased by self-selection of patients. PMP participants are generally more likely to take an active role in managing their own care and may already have been more motivated beforehand. Second, the participating group included those patients who participated only for part of the study duration. Therefore, the effects of the PMP suggested by the results of this study may underestimate the true magnitude of the impact of such programs. Third, regarding the economical outcome of the study the administrative cost of the care management program and the cost for the drug IM IFNb-1a itself has not been taken into account. Lastly, the effect of disease management may take years to become evident in patients’ health outcome and in the utilization of healthcare services [36]. Therefore this study, based on the relatively short follow-up period of one year, may miss long-term beneficial or detrimental effects or cost. Regarding the clinical outcomes, the observed reductions in relapse rates during treatment may have been affected by the regression to the mean phenomenon. Due to all these limitations, the results of the C.A.R.E. study should be regarded as preliminary and should be interpreted with due caution. Clearly, further research on the long-term effects of PMPs in MS patients is needed.

## Conclusions

In conclusion, in this observational study involving MS patients treated in with once weekly IM IFNb-1a routine care settings, participation in the optional patient management program MS-CARE was associated with apparently better therapeutic and economical outcomes in terms of improved medication adherence and lower total MS-related cost over time. As was shown in other therapeutic areas as well, structured patient management programs for MS patients may play a beneficial role in managing patient´s expectations and mitigating side-effects. PMPs may exert appreciable effects on treatment adherence and overall quality of care.
